# 3-Chloro-4-methyl­quinolin-2(1*H*)-one

**DOI:** 10.1107/S1600536812009889

**Published:** 2012-03-14

**Authors:** Mohamed G. Kassem, Hazem A. Ghabbour, Hatem A. Abdel-Aziz, Hoong-Kun Fun, Chin Wei Ooi

**Affiliations:** a Department of Pharmaceutical Chemistry, College of Pharmacy, King Saud University, PO Box 2457, Riyadh 11451, Saudi Arabia; b X-ray Crystallography Unit, School of Physics, Universiti Sains Malaysia, 11800 USM, Penang, Malaysia

## Abstract

The title compound, C_10_H_8_ClNO, is almost planar (r.m.s. deviation for the 13 non-H atoms = 0.023 Å). In the crystal, inversion dimers linked by pairs of N—H⋯O hydrogen bonds generate *R*_2_^2^(8) rings. Weak aromatic π–π stacking inter­actions [centroid–centroid distance = 3.7622 (12) Å] also occur.

## Related literature

For the biological activity of quinoline, see: Michael *et al.* (1996 ▶). For the synthesis, see: Hodgkinson & Staskun (1969 ▶). For hydrogen-bond motifs, see: Bernstein *et al.* (1995 ▶). For a related structure, see: Vasuki *et al.* (2001) ▶. For bond-length data, see: Allen *et al.* (1987 ▶).
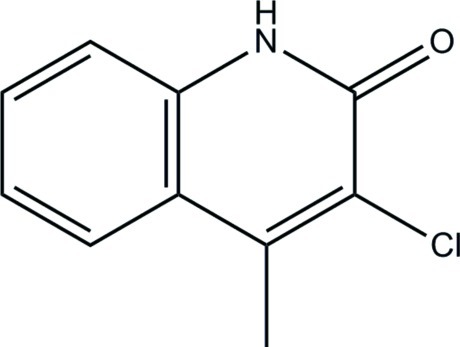


## Experimental

### 

#### Crystal data

C_10_H_8_ClNO
*M* *_r_* = 193.62
Monoclinic, 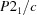

*a* = 3.9361 (2) Å
*b* = 12.9239 (6) Å
*c* = 17.1019 (7) Å
β = 100.197 (4)°
*V* = 856.23 (7) Å^3^
*Z* = 4
Cu *K*α radiation
μ = 3.56 mm^−1^
*T* = 296 K
0.92 × 0.10 × 0.10 mm

#### Data collection

Bruker APEXII CCD diffractometer
Absorption correction: multi-scan (*SADABS*; Bruker, 2009 ▶) *T*_min_ = 0.138, *T*_max_ = 0.720
5522 measured reflections
1434 independent reflections
1178 reflections with *I* > 2σ(*I*)
*R*_int_ = 0.040

#### Refinement

*R*[*F* ^2^ > 2σ(*F* ^2^)] = 0.037
*wR*(*F* ^2^) = 0.105
*S* = 1.00
1434 reflections
120 parameters
H-atom parameters constrained
Δρ_max_ = 0.18 e Å^−3^
Δρ_min_ = −0.21 e Å^−3^

### 

Data collection: *APEX2* (Bruker, 2009 ▶); cell refinement: *SAINT* (Bruker, 2009 ▶); data reduction: *SAINT*; program(s) used to solve structure: *SHELXTL* (Sheldrick, 2008 ▶); program(s) used to refine structure: *SHELXTL*; molecular graphics: *SHELXTL*; software used to prepare material for publication: *SHELXTL* and *PLATON* (Spek, 2009 ▶).

## Supplementary Material

Crystal structure: contains datablock(s) global, I. DOI: 10.1107/S1600536812009889/hb6671sup1.cif

Structure factors: contains datablock(s) I. DOI: 10.1107/S1600536812009889/hb6671Isup2.hkl

Supplementary material file. DOI: 10.1107/S1600536812009889/hb6671Isup3.cml

Additional supplementary materials:  crystallographic information; 3D view; checkCIF report

## Figures and Tables

**Table 1:** Hydrogen-bond geometry (Å, °)

*D*—H⋯*A*	*D*—H	H⋯*A*	*D*⋯*A*	*D*—H⋯*A*
N1—H1⋯O1^i^	0.93	1.91	2.816 (2)	166

Symmetry code: (i) 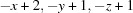.

**Fig. 1.**
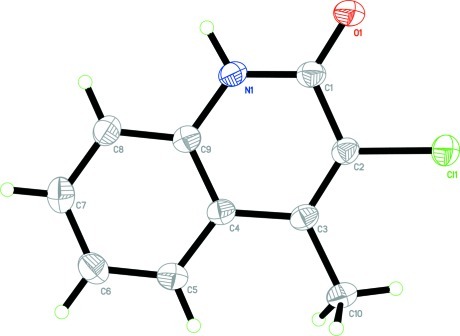
The molecular structure of the title compound, showing 30% probability displacement ellipsoids.

**Fig. 2.**
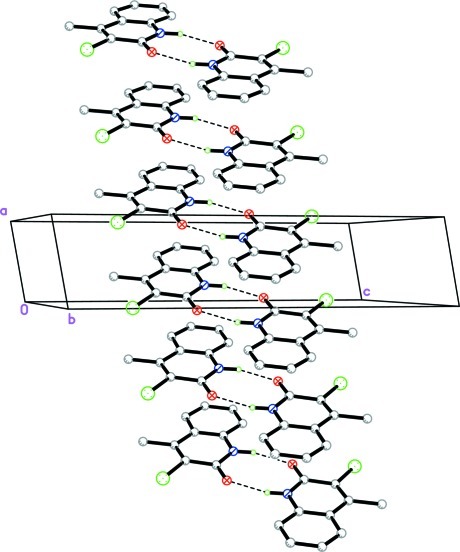
The crystal packing of the title compound, viewed along the b axis. H atoms not involved in the intermolecular interactions (dashed lines) have been omitted for clarity.

## supplementary crystallographic information

### Comment

For the previous reports of the chemistry and the biological activity of quinolines, see Michael *et al.* (1996).

In the title compound (Fig. 1), the quinoline ring (N1/C1–C9) is essentially planar with a maximum deviation of 0.012 (2) Å at atom C1. The bond lengths (Allen *et al.*, 1987) and angles are within normal ranges are comparable to the related structure (Vasuki *et al.*, 2001).

In the crystal structure (Fig. 2), the adjacent molecules are linked *via* pair of N1—H1···O1 (Table 1) hydrogen bonds, forming dimers with an *R*_2_^2^ (8) ring motif (Bernstein *et al.*, 1995). The crystal structure is further stabilized by weak π—π interactions between the benzene ring (*Cg*1; C4–C9) and quinoline ring (*Cg*2; N1/C1–C9). [*Cg*1···*Cg*2 = 3.7622 (12) Å; 1+*x*, y, *z*].

### Experimental

This compound was prepared according to the reported method (Hodgkinson & Staskun, 1969). Colorless needles of the title compound were grown from a mixed solution of EtOH/DMF (V/V = 2/1) by slow evaporation at room temperature.

### Refinement

Atom H1 was located from the difference map and was fixed at their found positions with *U*_iso_(H) = 1.2 *U*_eq_(N) [N–H = 0.9256 Å]. The remaining H atoms were positioned geometrically and refined using a riding model with *U*_iso_(H) = 1.2 or 1.5*U*_eq_(C) (C—H = 0.93 and 0.96 Å). A rotating group model was applied to the methyl group.

### Crystal data

**Table:**

C_10_H_8_ClNO	*F*(000) = 400
*M**_r_* = 193.62	*D*_x_ = 1.502 Mg m^−^^3^
Monoclinic, *P*2_1_/*c*	Cu *K*α radiation, λ = 1.54178 Å
Hall symbol: -P 2ybc	Cell parameters from 615 reflections
*a* = 3.9361 (2) Å	θ = 4.3–63.6°
*b* = 12.9239 (6) Å	µ = 3.56 mm^−^^1^
*c* = 17.1019 (7) Å	*T* = 296 K
β = 100.197 (4)°	Needle, colourless
*V* = 856.23 (7) Å^3^	0.92 × 0.10 × 0.10 mm
*Z* = 4	

### Data collection

**Table:**

Bruker APEXII CCD diffractometer	1434 independent reflections
Radiation source: fine-focus sealed tube	1178 reflections with *I* > 2σ(*I*)
Graphite monochromator	*R*_int_ = 0.040
φ and ω scans	θ_max_ = 64.9°, θ_min_ = 4.3°
Absorption correction: multi-scan (*SADABS*; Bruker, 2009)	*h* = −4→3
*T*_min_ = 0.138, *T*_max_ = 0.720	*k* = −15→14
5522 measured reflections	*l* = −20→17

### Refinement

**Table:**

Refinement on *F*^2^	Secondary atom site location: difference Fourier map
Least-squares matrix: full	Hydrogen site location: inferred from neighbouring sites
*R*[*F*^2^ > 2σ(*F*^2^)] = 0.037	H-atom parameters constrained
*wR*(*F*^2^) = 0.105	*w* = 1/[σ^2^(*F*_o_^2^) + (0.0755*P*)^2^] where *P* = (*F*_o_^2^ + 2*F*_c_^2^)/3
*S* = 1.00	(Δ/σ)_max_ = 0.001
1434 reflections	Δρ_max_ = 0.18 e Å^−^^3^
120 parameters	Δρ_min_ = −0.21 e Å^−^^3^
0 restraints	Extinction correction: *SHELXTL* (Sheldrick, 2008), Fc^*^=kFc[1+0.001xFc^2^λ^3^/sin(2θ)]^-1/4^
Primary atom site location: structure-invariant direct methods	Extinction coefficient: 0.0031 (9)

### Special details

**Table:**

Geometry. All e.s.d.'s (except the e.s.d. in the dihedral angle between two l.s. planes) are estimated using the full covariance matrix. The cell e.s.d.'s are taken into account individually in the estimation of e.s.d.'s in distances, angles and torsion angles; correlations between e.s.d.'s in cell parameters are only used when they are defined by crystal symmetry. An approximate (isotropic) treatment of cell e.s.d.'s is used for estimating e.s.d.'s involving l.s. planes.
Refinement. Refinement of *F*^2^ against ALL reflections. The weighted *R*-factor *wR* and goodness of fit *S* are based on *F*^2^, conventional *R*-factors *R* are based on *F*, with *F* set to zero for negative *F*^2^. The threshold expression of *F*^2^ > σ(*F*^2^) is used only for calculating *R*-factors(gt) *etc*. and is not relevant to the choice of reflections for refinement. *R*-factors based on *F*^2^ are statistically about twice as large as those based on *F*, and *R*- factors based on ALL data will be even larger.

### Fractional atomic coordinates and isotropic or equivalent isotropic displacement parameters (Å^2^)

**Table:**

	*x*	*y*	*z*	*U*_iso_*/*U*_eq_	
Cl1	1.04066 (14)	0.51266 (4)	0.76645 (3)	0.0516 (2)	
O1	1.0673 (4)	0.46152 (12)	0.60239 (9)	0.0560 (4)	
N1	0.7804 (4)	0.60196 (13)	0.54512 (9)	0.0422 (4)	
H1	0.7928	0.5780	0.4947	0.051*	
C1	0.9152 (5)	0.54435 (15)	0.60939 (12)	0.0414 (4)	
C2	0.8689 (5)	0.58782 (15)	0.68546 (11)	0.0386 (4)	
C3	0.7149 (4)	0.68001 (14)	0.69302 (11)	0.0371 (4)	
C4	0.5836 (5)	0.73765 (14)	0.62192 (11)	0.0368 (4)	
C5	0.4234 (5)	0.83478 (16)	0.62245 (12)	0.0446 (5)	
H5A	0.3935	0.8637	0.6706	0.054*	
C6	0.3104 (6)	0.88763 (17)	0.55307 (14)	0.0529 (6)	
H6A	0.2094	0.9525	0.5546	0.064*	
C7	0.3468 (6)	0.84441 (18)	0.48054 (14)	0.0552 (6)	
H7A	0.2690	0.8804	0.4337	0.066*	
C8	0.4965 (5)	0.74913 (17)	0.47760 (12)	0.0477 (5)	
H8A	0.5166	0.7198	0.4290	0.057*	
C9	0.6184 (5)	0.69643 (15)	0.54803 (11)	0.0385 (4)	
C10	0.6782 (6)	0.72335 (16)	0.77284 (11)	0.0468 (5)	
H10A	0.7537	0.6728	0.8133	0.070*	
H10B	0.4407	0.7403	0.7728	0.070*	
H10C	0.8168	0.7846	0.7833	0.070*	

### Atomic displacement parameters (Å^2^)

**Table:**

	*U*^11^	*U*^22^	*U*^33^	*U*^12^	*U*^13^	*U*^23^
Cl1	0.0660 (4)	0.0475 (3)	0.0416 (3)	0.0056 (2)	0.0105 (2)	0.0074 (2)
O1	0.0806 (11)	0.0439 (9)	0.0462 (9)	0.0171 (8)	0.0183 (7)	−0.0027 (7)
N1	0.0548 (10)	0.0398 (9)	0.0339 (9)	0.0011 (7)	0.0134 (7)	−0.0035 (7)
C1	0.0486 (11)	0.0363 (10)	0.0412 (10)	−0.0011 (8)	0.0129 (8)	−0.0036 (8)
C2	0.0443 (10)	0.0376 (10)	0.0351 (10)	−0.0038 (7)	0.0104 (7)	0.0000 (8)
C3	0.0386 (10)	0.0392 (10)	0.0349 (10)	−0.0065 (7)	0.0105 (7)	−0.0042 (8)
C4	0.0369 (10)	0.0363 (10)	0.0382 (10)	−0.0046 (7)	0.0090 (7)	−0.0041 (8)
C5	0.0451 (11)	0.0415 (11)	0.0474 (12)	−0.0002 (8)	0.0086 (8)	−0.0062 (9)
C6	0.0528 (12)	0.0430 (11)	0.0610 (14)	0.0065 (9)	0.0046 (10)	0.0014 (10)
C7	0.0574 (13)	0.0547 (14)	0.0506 (13)	0.0009 (10)	0.0014 (10)	0.0121 (10)
C8	0.0559 (12)	0.0498 (12)	0.0374 (11)	−0.0005 (9)	0.0084 (8)	0.0017 (9)
C9	0.0395 (10)	0.0389 (10)	0.0380 (10)	−0.0045 (7)	0.0096 (7)	−0.0026 (8)
C10	0.0551 (12)	0.0491 (12)	0.0380 (10)	0.0024 (9)	0.0128 (8)	−0.0080 (9)

### Geometric parameters (Å, º)

**Table:**

Cl1—C2	1.728 (2)	C5—C6	1.373 (3)
O1—C1	1.243 (3)	C5—H5A	0.9300
N1—C1	1.355 (3)	C6—C7	1.391 (3)
N1—C9	1.382 (3)	C6—H6A	0.9300
N1—H1	0.9256	C7—C8	1.370 (3)
C1—C2	1.458 (3)	C7—H7A	0.9300
C2—C3	1.353 (3)	C8—C9	1.393 (3)
C3—C4	1.442 (3)	C8—H8A	0.9300
C3—C10	1.506 (2)	C10—H10A	0.9600
C4—C9	1.400 (3)	C10—H10B	0.9600
C4—C5	1.406 (3)	C10—H10C	0.9600
			
C1—N1—C9	124.95 (17)	C5—C6—C7	120.2 (2)
C1—N1—H1	119.6	C5—C6—H6A	119.9
C9—N1—H1	115.4	C7—C6—H6A	119.9
O1—C1—N1	121.42 (18)	C8—C7—C6	120.4 (2)
O1—C1—C2	123.80 (19)	C8—C7—H7A	119.8
N1—C1—C2	114.78 (17)	C6—C7—H7A	119.8
C3—C2—C1	123.60 (18)	C7—C8—C9	119.5 (2)
C3—C2—Cl1	122.45 (15)	C7—C8—H8A	120.3
C1—C2—Cl1	113.93 (15)	C9—C8—H8A	120.3
C2—C3—C4	118.30 (17)	N1—C9—C8	119.43 (18)
C2—C3—C10	122.04 (18)	N1—C9—C4	119.21 (18)
C4—C3—C10	119.65 (17)	C8—C9—C4	121.37 (19)
C9—C4—C5	117.48 (18)	C3—C10—H10A	109.5
C9—C4—C3	119.13 (18)	C3—C10—H10B	109.5
C5—C4—C3	123.38 (18)	H10A—C10—H10B	109.5
C6—C5—C4	121.0 (2)	C3—C10—H10C	109.5
C6—C5—H5A	119.5	H10A—C10—H10C	109.5
C4—C5—H5A	119.5	H10B—C10—H10C	109.5
			
C9—N1—C1—O1	−177.64 (19)	C9—C4—C5—C6	−0.9 (3)
C9—N1—C1—C2	1.9 (3)	C3—C4—C5—C6	178.36 (19)
O1—C1—C2—C3	177.3 (2)	C4—C5—C6—C7	1.4 (3)
N1—C1—C2—C3	−2.2 (3)	C5—C6—C7—C8	−0.3 (3)
O1—C1—C2—Cl1	−0.8 (3)	C6—C7—C8—C9	−1.2 (3)
N1—C1—C2—Cl1	179.65 (14)	C1—N1—C9—C8	178.74 (18)
C1—C2—C3—C4	1.2 (3)	C1—N1—C9—C4	−0.6 (3)
Cl1—C2—C3—C4	179.19 (13)	C7—C8—C9—N1	−177.72 (19)
C1—C2—C3—C10	−178.76 (18)	C7—C8—C9—C4	1.6 (3)
Cl1—C2—C3—C10	−0.7 (3)	C5—C4—C9—N1	178.79 (17)
C2—C3—C4—C9	0.2 (3)	C3—C4—C9—N1	−0.5 (3)
C10—C3—C4—C9	−179.84 (16)	C5—C4—C9—C8	−0.5 (3)
C2—C3—C4—C5	−179.06 (18)	C3—C4—C9—C8	−179.88 (17)
C10—C3—C4—C5	0.9 (3)		

### Hydrogen-bond geometry (Å, º)

**Table:**

*D*—H···*A*	*D*—H	H···*A*	*D*···*A*	*D*—H···*A*
N1—H1···O1^i^	0.93	1.91	2.816 (2)	166

Symmetry code: (i) −*x*+2, −*y*+1, −*z*+1.
